# Electrolyte imbalances in an unselected population in an emergency department: A retrospective cohort study

**DOI:** 10.1371/journal.pone.0215673

**Published:** 2019-04-25

**Authors:** Kiarash Tazmini, Ståle H. Nymo, William E. Louch, Anette H. Ranhoff, Erik Øie

**Affiliations:** 1 Department of Internal Medicine, Diakonhjemmet Hospital, Oslo, Norway; 2 Institute of Experimental Medical Research, Oslo University Hospital, Ullevål and University of Oslo, Oslo, Norway; 3 Center for Heart Failure Research, University of Oslo, Oslo, Norway; 4 Department of Clinical Science, University of Bergen, Bergen, Norway; Azienda Ospedaliero Universitaria Careggi, ITALY

## Abstract

**Background:**

Although electrolyte imbalances (EIs) are common in the emergency department (ED), few studies have examined the occurrence of such conditions in an unselected population.

**Objectives:**

To investigate the frequency of EI among adult patients who present to the ED, with regards to type and severity, and the association with age and sex of the patient, hospital length of stay (LOS), readmission, and mortality.

**Methods:**

A retrospective cohort study. All patients **≥**18 years referred for any reason to the ED between January 1, 2010, and December 31, 2015, who had measured blood electrolytes were included. In total, 62 991 visits involving 31 966 patients were registered.

**Results:**

EIs were mostly mild, and the most common EI was hyponatremia (glucose-corrected) (24.6%). Patients with increasing severity of EI had longer LOS compared with patients with normal electrolyte measurements. Among all admitted patients, there were 12928 (20.5%) readmissions within 30 days from discharge during the study period. Hyponatremia (glucose-corrected) was associated with readmission, with an adjusted odds ratio (OR) of 1.25 (95% CI, 1.18–1.32). Hypomagnesemia and hypocalcemia (albumin-corrected) were also associated with readmission, with ORs of 1.25 (95% CI, 1.07–1.45) and 1.22 (95% CI, 1.02–1.46), respectively. Dysnatremia, dyskalemia, hypercalcemia, hypermagnesemia, and hyperphosphatemia were associated with increased in-hospital mortality, whereas all EIs except hypophosphatemia were associated with increased 30-day and 1-year mortality.

**Conclusions:**

EIs were common and increasing severity of EIs was associated with longer LOS and increased in-hospital, 30-days and 1-year mortality. EI monitoring is crucial for newly admitted patients, and up-to-date training in EI diagnosis and treatment is essential for ED physicians.

## Introduction

Electrolyte imbalance (EI) is common in hospitalized patients as well as in the general population and is associated with increased morbidity and mortality [1–9]. Clinically important EIs include dysnatremia, dyskalemia, dyscalcemia, dysmagnesemia, and dysphosphatemia. The prevalence of hyponatremia in the emergency department (ED) is reported to range from 2.3–44%, while prevalence of hypernatremia is 1.1–4.4%, hypokalemia 10.2–39%, hyperkalemia 0.8–13%, and albumin-corrected hypercalcemia 0.7–7.5% [1, 2, 6, 10–13].

EIs have previously been investigated in several different cohorts. However, most previous studies have investigated one or two specific electrolytes in a selected group of patients with a single disease (e.g., heart or kidney disease) [14, 15], or in patients in a particular risk group (e.g., intensive care patients or patients using diuretics) [16, 17]. Previous studies have shown an association between EI and increased hospital length of stay (LOS) [8, 9, 13, 18–26] and a correlation between hyponatremia and rate of readmission [18–20, 27]. Although EI is frequently found in clinical practice, there are few studies which have examined frequency and outcomes in an unselected group of adult patients admitted to the ED. Apart from some studies of hyponatremia and readmission [18–20, 27], the frequency of readmissions among patients with other EIs is unknown. Data are particularly sparse regarding outcomes of dyscalcemia, dysmagnesemia, and dysphosphatemia.

We aimed to investigate the prevalence and severity of sodium and potassium imbalances among all adult patients visiting the ED, as well as imbalances of albumin-corrected calcium, free calcium, magnesium and phosphate levels in patients where these electrolytes were measured. We also examined possible associations between EIs and patient age and sex and with the clinically important outcomes LOS, readmission, in-hospital mortality and mortality 30 days and one year after discharge.

## Methods

### Study population, design and data source

All patients 18 years or older referred for any reason to the ED at Diakonhjemmet Hospital between January 1, 2010, and December 31, 2015, were included in this retrospective cohort study. During the study period, 62 991 patient contacts were registered, involving 31 966 unique patients with laboratory blood analyses (S1 Fig). Diakonhjemmet Hospital is a local urban hospital in Oslo, Norway, which serves approximately 135 000 inhabitants. Patients are referred to the ED from general practitioners, municipal emergency services, nursing homes, community health services or directly by ambulance ordered from a dispatch center. For every visit to the ED, age, sex, patient category (medical or surgical), serum-electrolyte values and serum-albumin and glucose levels were registered.

Serum-sodium levels were corrected for serum-glucose by lowering the sodium concentration by 2.4 mmol/L for every 5.5 mmol/L increase in glucose (glucose-corrected serum-sodium = serum-sodium + [(serum-glucose– 5,6)/5,6] x 2,4), to account for the diluting effect of hyperglycemia [28]. The reference range for glucose according to our hospital’s laboratory was 4.0–6.0 mmol/L. A correction formula was also used to calculate albumin-corrected calcium levels (mmol/L) [= measured serum-calcium level + 0.020 × (41.3 –serum-albumin) where 41.3 g/L is the albumin median] [29].

At Diakonhjemmet Hospital, measurement of serum levels of sodium, potassium, calcium, albumin and glucose are performed routinely in all medical patients in the ED (serum levels of calcium, albumin and glucose are not measured in surgical patients), whereas measurement of serum-magnesium, -phosphate and plasma-free calcium are carried out when indicated. We registered EIs for every ED visit.

We also recorded LOS, readmission within 30 days post-discharge, and in-hospital, 30-day, and 1-year mortality. Primary and secondary diagnoses as classified by the International Classification of Diseases, 10^th^ revision (ICD-10), were registered. Data were extracted from the hospital’s Department of Medical Biochemistry database and the patient administrative system. After obtaining the data, the electrolyte values were categorized into groups based on the severity of imbalance (mild, moderate and severe) according to Table 1.

**Table 1:** The reference range *, and definition of electrolyte imbalances by degree of severity. All values are in conventional units with SI units in parentheses.

Electrolyte	Ref. range	Hypo-			Hyper-		
		Mild	Moderate	Severe	Mild	Moderate	Severe
**S-sodium** **mmol/L**	137–145	130–136	125–129	<125	146–154	155–165	>165
**S-potassium mmol/L**	3.6–5.0	3.0–3.5	2.5–2.9	<2.5	5.1–5.9	6.0–6.9	≥7.0
**S-Albumin-corrected- calcium mmol/L**	2.15–2.51	1.90–2.14	1.65–1.89	<1.65	2.52–2.75	2.76–3.00	3.01–3.50
**P-free-calcium mmol/L**	1.14–1.28	1.00–1.13	0.80–0.99	<0.80	1.29–1.50	1.51–1.70	1.70–2.00
**S-magnesium** **mmol/L**	0.71–0.94	0.66–0.70	0.50–0.65	<0.5o	0.95–2.0	2.10–5.00	>5.00
**S-phosphate** **mmol/L**	Female 0.85–1.50 Male 18–49 yrs 0.75–1.65 Male > 49 yrs 0.75–1.35	Female 0.65–0.84 Male 0.65–0.74	0.30–0.64	<0.30	Female 1.66–1.74 Male 18–49 yrs 1.66–1.74 Male > 49 yrs 1.36–1.74	1.75–2.00	>2.00

* According to our hospitals laboratory which applies SI units.

The study was evaluated by the regional committee for medical and health research ethics (Regional Committee for Medical and Health Research Ethics South East) and defined as a quality study. It was also approved by the institutional review board (The Research Committee, Diakonhjemmet Hospital). Since all data were fully anonymized before we accessed them, the regional committee for medical research ethics and the institutional review board waived the requirement for informed consent.

### Statistical analyses

The results are analyzed and presented in relation to each ED visit, not patient, since many patients had more than one visit during the study period. The results are presented as median with interquartile range (IQR) for continuous data, and as percentages for categorical data. Univariable and multivariable logistic regression analyses were performed to study associations between EI and readmissions, in-hospital mortality, and 30-day mortality. Cox proportional hazard models were performed to analyze the association between EI and 1-year mortality. The multivariable analyses were adjusted for age, sex and the comorbid conditions (hypertension, heart failure, chronic obstructive pulmonary disease, cancer, kidney failure, diabetes mellitus, atrial fibrillation, pneumonia, sepsis, dehydration, and hip fracture). A two-sided *P*-value less than 0.05 was considered significant. Statistical analyses were performed using Stata/SE (version 14.2; Stata Corporation, College Station, TX).

## Results

### Baseline characteristics

The median age of all patients was 69 years (IQR 51–82 years). 33 705 patients (53.5%) were female, 44 381 (70.8%) were referred for medical reasons, and 18 325 (29.2%) were referred for surgery. Most of the patients were older (S2 Fig); 12 005 (19.1%) were 40–59 years, 21 970 (35.0%) were 60–79 years, and 19 146 (30.5%) were 80 years and older.

Electrolyte imbalances were mostly mild, and the most common EI was hyponatremia (glucose-corrected) (24.6%) (Table 2).

**Table 2:** Electrolyte imbalance frequency by degree of severity for all ED visits 2010–2015.

	Proportion (%)	Mild n (%)	Moderate n (%)	Severe n (%)
**Sodium**				
Hyponatremia	15 030/62 929 (23.9)	13 186 (87.7)	1350 (9.0)	494 (3.3)
Hypernatremia	1076/62 929 (1.7)	989 (91.9)	69 (6.4)	18 (1.7)
**Glucose corrected sodium**				
Hyponatremia	11 692/47 467 (24.6)	10 224 (87.4)	1051 (9.0)	417 (3.6)
Hypernatremia	1894/47 467 (4.0)	1781 (94.0)	81 (4.3)	32 (1.7)
**Potassium**				
Hypokalemia	5376 /62 730 (8.6)	4931 (91.7)	396 (7.4)	49 (0.9)
Hyperkalemia	2080/62 730 (3.3)	1834 (88.2)	197 (9.5)	49 (2.4)
**Calcium (albumin-corrected)**				
Hypocalcemia	713/45 675 (1.6)	657 (92.2)	40 (5.6)	16 (2.2)
Hypercalcemia	4981/45 675 (10.9)	4527 (90.9)	341 (6.9)	113 (2.3)
**Calcium (free)**				
Hypocalcemia	3559/14 835 (24.0)	3454 (97.1)	97 (2.7)	8 (0.20)
Hypercalcemia	555/14 835 (3.7)	501 (90.3)	41 (7.4)	13 (2.3)
**Magnesium**				
Hypomagnesemia	1226/8512 (14.4)	594 (48.5)	563 (46.0)	69 (5.6)
Hypermagnesemia	691/8512 (8.1)	691 (100)	0	0
**Phosphate**				
Hypophosphatemia	715/7621 (9.4)	533 (74.6)	174 (24.3)	8 (1.1)
Hyperphosphatemia	634/7621 (8.3)	357 (56.3)	116 (18.3)	161 (25.4)

Abbreviations: ED, Emergency Department.

Hyponatremia, hypokalemia, hypercalcemia, dysmagnesemia, and hypophosphatemia were more frequent among female patients (S1 Table).

Among all visits (62 991), all electrolytes were measured in 7438 (11,8%), and among these visits, there were 2783 (37.4%) who had no EI, 2546 (34.2%) had one EI, 1290 (17.3%) had two EIs, and 819 (11%) had three or more EIs. Furthermore, among all visits who had measured sodium, potassium, and albumin-corrected calcium (45 500), 19207 (42,2%) had at least one of these EIs. The median serum-glucose levels for all ED-visits was 6.2 (5.5–7.4) mmol/L, and there were 26 303 (55.4%) visits with serum-glucose > 6.0 mmol/L. Among ED visits with hyperglycemia (s-glucose > 6.0 mmol/L), 10 905 (26.1%) had hyponatremia and 672 (1.6%) hypernatremia.

### Hospital length of stay

18 541 (29.4%) patients were discharged from the ED and not admitted. The median LOS among admitted patients was 3 days (IQR 1–5 days). While 31 062 (49.3%) of the patients were admitted for 1–4 days, 13 388 (21.3%) were admitted for 5 days or longer. Patients with increasing severity of EI had longer LOS compared with patients with normal electrolyte measurements (Table 3).

**Table 3:** Median length of hospital stay by degree of severity of the electrolyte imbalance and number of readmissions within 30 days after discharge for all admissions 2010–2015.

	Normo- days (IQR)	Mild days (IQR)	Moderate days (IQR)	Severe days (IQR)	Readmitted n (%)
**Sodium**					
Normonatremia (n = 31 154)	2 (1–5)				9169 (19.6)
Hyponatremia (n = 12 125)		3 (2–6)	4 (2–8)	5 (3–8)	3557 (23.6)
Hypernatremia (n = 854)		3 (1–7)	5 (3–9)	6 (3–11)	187 (17.4)
**Glucose corrected sodium**					
Normonatremia (n = 23 490)	2 (1–5)				6319 (18.7)
Hyponatremia (n = 9479)		3 (2–6)	4 (2–7)	5 (3–8)	2693 (23.0)
Hypernatremia (n = 1521)		3 (2–6)	4 (3–8)	6 (4–10)	381 (20.1)
**Potassium**					
Normokalemia (n = 38 241)	2 (1–5)				11 352 (20.5)
Hypokalemia (n = 4351)		3 (2–6)	5 (2–9)	6 (2–11)	1023 (19.0)
Hyperkalemia (n = 1663)		4 (2–7)	4 (2–7)	4 (1–10)	500 (24.2)
**Calcium (albumin-corrected)**					
Normocalcemia (n = 28 746)	3 (1–5)				7781 (19.5)
Hypocalcemia (n = 546)		4 (2–7)	4 (2–7)	7.5 (2–11)	164 (23.0)
Hypercalcemia (n = 4063)		3 (1–6)	3 (1–7)	5 (3–11)	1111 (22.3)
**Calcium (free)**					
Normocalcemia (n = 8430)	3 (1–6)				2003 (18.7)
Hypocalcemia (n = 3056)		4 (2–7)	5 (2–7)	6.5 (2–10)	739 (20.8)
Hypercalcemia (n = 472)		4 (2–7)	5 (2–11)	9.5 (4–13.5)	116 (20.9)
**Magnesium**					
Normomagnesemia (n = 5778)	4 (2–7)				1193 (18.1)
Hypomagnesemia (n = 1135)		4 (2–7)	4 (2–7)	4 (2–7)	264 (21.6)
Hypermagnesemia (n = 622)		4 (2–8)	NA	NA	133 (19.3)
**Phosphate**					
Normophosphatemia (n = 5563)	4 (2–7)				1151 (18.4)
Hypophosphatemia (n = 658)		4 (2–8)	4 (2–7)	3 (2–11)	122 (17.1)
Hyperphosphatemia (n = 580)		4 (2–9)	6 (3–10.5)	4 (2–10)	125 (19.8)

Abbreviations: NA, not applicable, IQR, inter quartile range.

### Readmission

Among all admitted patients, there were 12 928 (20.5%) readmissions within 30 days from discharge during the study period. Patients with hyponatremia accounted for 3557 (23.6%) of these readmissions (Table 3). Among those who were not readmitted, 27 170 (54.3%) were females and the median age was 68 years (IQR, 49–82 years). By comparison, among those who were readmitted, 6 535 (50.6%) were females and the median age was 71 (IQR 57–82 years). The median LOS was 1 day (IQR 1–4 days) for both groups. EIs were not significantly different between patients who were not readmitted compared with those who were readmitted, whereas 30-days mortality was higher in patients who were readmitted (40%) compared with patients who were not readmitted (26.5%). Hyponatremia (glucose-corrected) was associated with readmission with an adjusted odds ratio (OR) of 1.25 (95% CI, 1.18–1.32). Similar associations were observed for hypomagnesemia and hypocalcemia (albumin-corrected calcium) with ORs of 1.25 (95% CI, 1.07–1.45) and 1.22 (95% CI, 1.02–1.46) (Table 4). Among all admissions with mild EIs the readmission rate was 10–20%, whereas it was 0.2–10% for patients with moderate to severe EIs (S2 Table). Increasing severity of EIs was not associated with an elevated OR for readmission (S3 Table).

**Table 4:** Multivariate analysis for readmission, in-hospital, 30-day and 1-year mortality for all ED visits 2010–2015.

	Multivariable analysis readmission*		Multivariable analysis in-hospital mortality^a^		Multivariable analysis 30-days mortality^a^		Multivariable analysis 1-year mortality^a^	
	Odds ratio (95% CI)	p-value	Odds ratio (95% CI)	p-value	Odds ratio (95% CI)	p-value	Hazard ratio (95% CI)	p-value
**Normonatremi (reference)**								
Hyponatremia	1.19 (1.14–1.25)	<0.0001	1.58 (1.40–1.78)	<0.0001	1.25 (1.21–1.29)	<0.0001	1.46 (1.39–1.54)	<0.0001
Hypernatremia	0.82 (0.70–0.96)	0.016	4.66 (3.72–5.82)	<0.0001	1.62 (1.46–1.80)	<0.0001	1.79 (1.54–2.08)	<0.0001
**Glucose corrected normonatremi (reference)**								
Hyponatremia	1.25 (1.18–1.32)	<0.0001	1.53 (1.34–1.74)	<0.0001	1.24 (1.19–1.28)	<0.0001	1.45 (1.37–1.53)	<0.0001
Hypernatremia	1.01 (0.90–1.14)	0.802	3.69 (3.06–4.46)	<0.0001	1.40 (1.30–1.52)	<0.0001	1.59 (1.42–1.79)	<0.0001
**Normokalemia (reference)**								
Hypokalemia	0.93 (0.86–1.00)	0.042	1.73 (1.46–2.06)	<0.0001	1.20 (1.14–1.26)	<0.0001	1.25 (1.15–1.35)	<0.0001
Hyperkalemia	1.09 (0.98–1.21)	0.126	3.33 (2.81–3.95)	<0.0001	1.45 (1.35–1.55)	<0.0001	1.54 (1.39–1.70)	<0.0001
**Normocalcemia (albumin-corrected calcium, reference)**								
Hypocalcemia	1.22 (1.02–1.46)	0.032	1.38 (0.91–2.09)	0.126	1.42 (1.25–1.60)	<0.0001	1.52 (1.27–1.81)	<0.0001
Hypercalcemia	1.06 (0.99–1.14)	0.119	2.35 (2.05–2.70)	<0.0001	1.45 (1.38–1.51)	<0.0001	1.70 (1.59–1.82)	<0.0001
**Normocalcemia (free-calcium)**								
Hypocalcemia	1.11 (1.00–1.22)	0.037	1.10 (0.92–1.31)	0.281	1.09 (1.03–1.17)	0.005	1.14 (1.03–1.26)	<0.009
Hypercalcemia	1.00 (0.81–1.25)	0.947	2.10 (1.57–2.80)	<0.0001	1.43 (1.25–1.64)	<0.0001	1.61 (1.33–1.95)	<0.0001
**Normomagnesemia (reference)**								
Hypomagnesemia	1.25 (1.07–1.45)	0.004	1.02 (0.74–1.40)	0.919	1.12 (1.03–1.22)	0.010	1.29 (1.12–1.48)	<0.0001
Hypermagnesemia	1.10 (0.90–1.35)	0.353	2.45 (1.83–3.28)	<0.0001	1.13 (1.00–1.27)	0.046	1.27 (1.06–1.52)	0.009
**Normophosphatemia (reference)**								
Hypophosphatemia	0.95 (0.77–1.16)	0.601	0.82 (0.53–1.26)	0.368	0.94 (0.84–1.04)	0.226	0.76 (0.63–0.94)	0.009
Hyperphosphatemia	1.08 (0.87–1.34)	0.498	3.80 (2.86–5.06)	<0.0001	1.29 (1.13–1.48)	<0.0001	1.37 (1.13–1.67)	0.001

Abbreviations: ED, Emergency Department.

* Adjusted for age, sex and comorbid conditions (hypertension, heart failure, atrial fibrillation/atrial flutter, chronic pulmonary disease, cancer, kidney failure, dehydration, diabetes mellitus, pneumonia, sepsis and hip fracture).

#### In-hospital, 30-day and 1-year mortality

**Fig 1:**
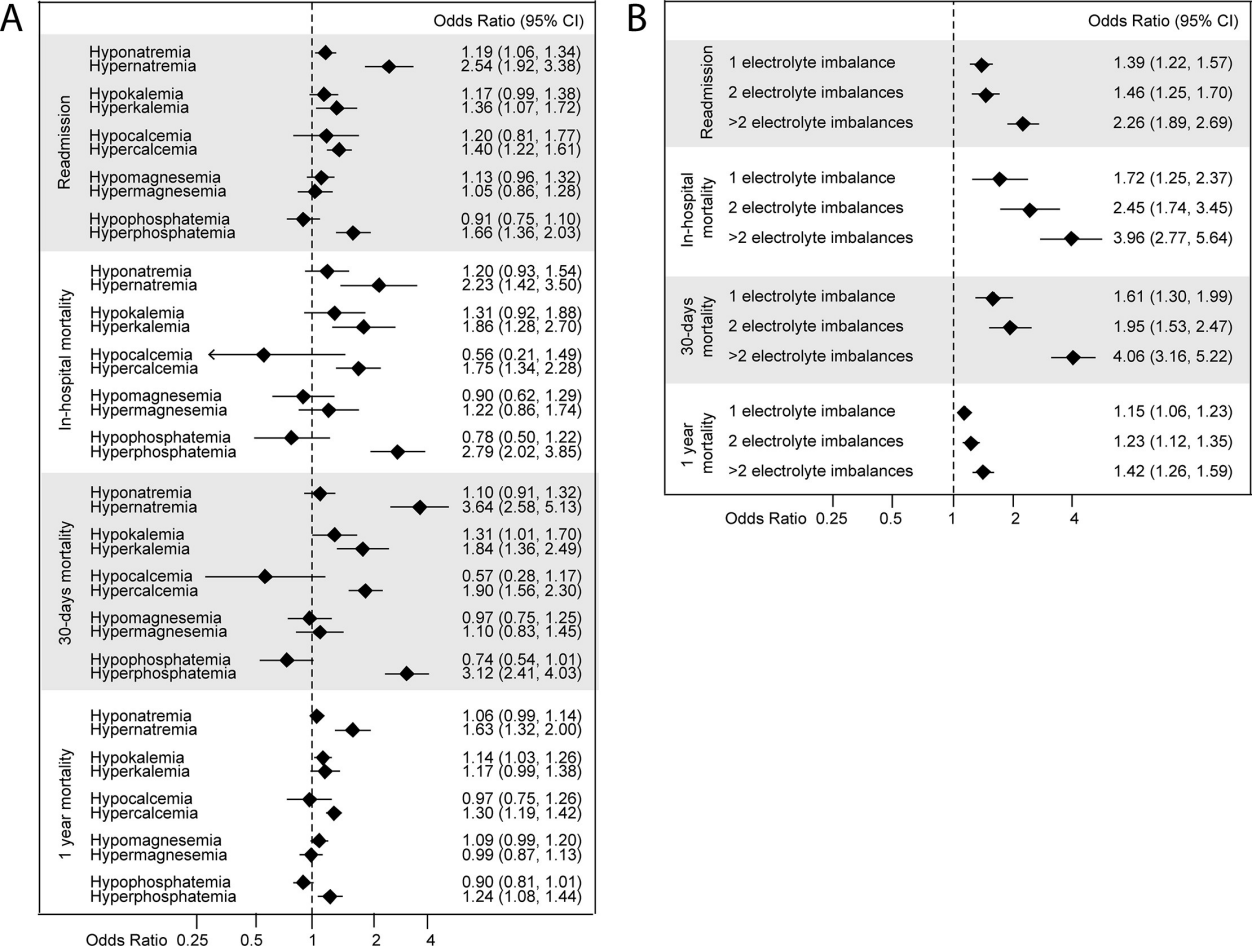
In-hospital, 30-days, 1-year mortality and readmission. **A.** Adjusted for all electrolyte imbalances, age, sex and comorbid conditions (hypertension, heart failure, atrial fibrillation/atrial flutter, chronic pulmonary disease, cancer, kidney failure, dehydration, diabetes mellitus, pneumonia, sepsis and hip fracture). **B.** Adjusted after number of electrolyte imbalances; 1, 2, or >2 electrolyte imbalances.

Dysnatremia, dyskalemia, hypercalcemia, hypermagnesemia, and hyperphosphatemia were associated with increased in-hospital mortality, and all EIs except hypophosphatemia were associated with increased 30-day and 1-year mortality compared with patients not exhibiting the specific EI (Table 4). After adjusting for other EIs and comorbidities, hypernatremia, hyperkalemia, hypercalcemia, and hyperphosphatemia were still associated with increased in-hospital mortality and 30-day mortality. Hypernatremia, hypercalcemia, and hyperphosphatemia were associated with increased 1-year mortality (Fig 1).

## Discussion

This is the first study of a non-selected, adult ED patient population describing the prevalence of EI according to type, severity, and associations to outcomes such as LOS, readmission, and mortality. Nearly half of the patients exhibited at least one type of EI, but most were mild. Increasing severity of EI was associated with longer LOS, increased in-hospital, 30-day and 1-year mortality.

### Prevalence of EIs

In our patient population, we observed a high prevalence of hyponatremia (24.6% of patients, glucose-corrected sodium), frequent hypokalemia and albumin-corrected hypercalcemia (8.6% and 10.9%, respectively), and lower prevalence of hypernatremia, hyperkalemia, and albumin-corrected hypocalcemia (1.7%, 3.3%, and 1.6%, respectively). These findings are in line with previous prevalence studies in ED which have shown prevalence of hyponatremia to be 2.3–44%, hypernatremia 1.1–4.4%, hypokalemia 10.2–39%, hyperkalemia 0.8–13%, and albumin-corrected hypercalcemia 0.7–7.5% [1, 2, 6, 10–13]. The large variation in the reported prevalence of these EIs is likely related to differences in the threshold used to define the imbalances, the time of the measurement (e.g., at admission, during hospitalization), and the study population (i.e. young vs. older patients). Most of our patients with EI were older (>60 years of age), suggesting that aging is an important determinant for developing EI. Indeed, impairment of renal function and changes in neurohumoral homeostasis during aging are well established. In addition, medical conditions and polypharmacy which are more prevalent in older people likely contribute to EI in this population [30].

The prevalence of abnormal levels of magnesium and phosphate in our overall patient cohort is unknown since these two electrolytes were only measured when indicated by the attending physician. When measured, patients with magnesium and phosphate values in the normal range had a median LOS of 4 days, compared with only 2 days in the unselected population of patients with sodium and potassium values in normal range. A probable explanation for this observation is that magnesium and phosphate levels are measured in patients with malnutrition, weight loss, arrhythmias, renal failure, and other EIs as well as in the oldest patients. Among patients presenting with dysmagnesemia and dysphosphatemia, 14.4% exhibited hypomagnesemia, 8.1% hypermagnesemia, 9.4% hypophosphatemia, and 8.3% hyperphosphatemia, and these patients accounted for 25.6% of ED visits.

Because of the diluting effect of hyperglycemia on sodium concentration, the true value of s-sodium in hyperglycemic patients is glucose-corrected sodium [28]. One previous study demonstrated the superiority of glucose-corrected serum-sodium to predict mortality over measured serum-sodium, and the authors suggested that glucose-corrected serum-sodium should be considered in studies analyzing serum-sodium [31]. To our knowledge our study is the first study to report serum-sodium and glucose-corrected serum-sodium in an unselected hospital population.

Since most of our patients had serum-calcium and serum-albumin levels analyzed, albumin-corrected calcium could be calculated. However, in as many as 23.6% of the ED visits free-calcium was analyzed in plasma. Of these, 24.0% of patients exhibited hypocalcemia and 3.7% hypercalcemia. In comparison with albumin-corrected calcium levels, hypercalcemia was less frequently observed when measured as free calcium in plasma. Conversely, hypocalcemia was more frequent when measured as plasma-free calcium. This discrepancy could be due to a selection of patients where arterial blood was drawn for blood gas analysis, in addition to absence of both hemolysis and venous stasis which can lead to an increase in calcium. Therefore, it seems like albumin-corrected calcium measurements overestimates calcium levels. Similar results have been previously reported in a study investigating blood gas analyses in patients visiting the ED (25.3% hypocalcemia and 3% had hypercalcemia) [32]. Numerous studies have identified specific clinical situations in which direct measurement of free-calcium has been shown to be superior to its calculation from total calcium and albumin, even with corrections for pH [33]. Thus, in our study, the results from free-calcium may be closer to the actual dyscalcemia than albumin-corrected calcium. To our knowledge, the present study is the first to compare albumin-corrected with free calcium in an unselected population admitted to the ED.

### Hospital length of stay

It is well known that dysnatremia is associated with longer LOS. According to a recent meta-analysis investigating 46 studies, mean LOS was 3 days longer in hyponatremic patients[18]. In another recent study, LOS was 10% longer for patients with community-acquired hypernatremia [34]. In our study, the median LOS for patients with mild, moderate, and severe hyponatremia and hypernatremia was 3, 4, and 5 days and 3, 5, and 6 days, respectively, compared with 2 days for normonatremic patients. Our results demonstrate a similar increase in LOS for patients with hypokalemia as patients with dysnatremia, with median hospital stays of 3, 5, and 6 days for mild, moderate, and severe hypokalemia. For hyperkalemia, median LOS was 4, 4, and 4 days, respectively. Of note, the number of patients with severe dyskalemia was very low. Importantly, while the median LOS is perhaps shorter than expected in patients with severe hyperkalemia, it should be noted that most of these patients likely suffered from severe renal failure and thus were transferred to the nearby university hospital for dialysis. High mortality in this patient group also likely reduced LOS values. We are aware of only a single retrospective study of potassium levels in ED patients, which reported a mean LOS of 5.8 days for patients with hypokalemia and 6.6 days for patients with hyperkalemia. However, in this study, the mean LOS for normokalemic patients was substantially longer than at our hospital (4.8 days) [9].

In our study, the median LOS for patients with severe hypocalcemia was 6.5 days when measured as free calcium and 7.5 days when measured as albumin-corrected calcium, respectively. For patients with severe hypercalcemia, the median LOS was 9.5 days when measured as free calcium and 5 days when measured as albumin-corrected calcium, respectively. Thus, these patients had significantly longer hospital stays than normocalcemic patients, for whom median LOS was 3 days. An earlier study reported a mean LOS of 10 days in patients with hypercalcemia, mirroring our results. In this previous study, however, the mean LOS was much longer for all patients than in our study, and there was no significant correlation between serum-calcium level and the length of hospital stay [13].

Although the population of patients where magnesium and phosphate were measured was likely more morbid, median LOS was only slightly increased for those exhibiting dysmagnesemia and hypophosphatemia compared with normomagnesemia and normophosphatemia. Patients with moderate hyperphosphatemia, however, exhibited longer LOS in comparison with normophosphatemic patients (median LOS = 6 vs 4 days). This difference may at least partly reflect renal failure, which is a common cause of hyperphosphatemia. This increase in LOS was lower in patients with severe hyperphosphatemia compared with patients with moderate hyperphosphatemia, again possibly reflecting transfer of patients with severe hyperphosphatemia to the nearby university hospital for dialysis. Indeed, a previous retrospective study investigating ED patients found a substantial reduction of renal function in patients with hyperphosphatemia upon admission[26]. In parallel to our findings, a mean LOS of 6 days was previously reported in patients with hyperphosphatemia compared with 3 days for patients with normophosphatemia [26].

### Readmission

In a multivariate analysis adjusted for age, sex and comorbid conditions, only hyponatremia, hypocalcemia, and hypomagnesemia were significantly associated with increased probability for readmission. Hypernatremia was not associated with increased probability for readmission due to the high in-hospital mortality for these patients. A similar lack of increased OR for readmission with increasing severity of EIs likely also reflects higher mortality in patients with the most severe EIs.

### Mortality

In our study, hyponatremia and hypokalemia were associated with 58% and 73% increases in risk of in-hospital mortality, but such associations were not observed for hypocalcemia, hypomagnesemia, and hypophosphatemia. However, for all electrolytes increased levels were associated with a substantial increase in-hospital mortality (2.1–4.6-fold increase). For all EIs except dysphosphatemia, the risk of mortality increased with greater EI severity. Except for hypophosphatemia, all other EIs (decreased and increased electrolyte levels) were associated with increased 30-day and 1-year mortality values. However, this association was much more marked when electrolyte levels were *increased*, as 30-day and 1-year mortality values were elevated by 13–79%. These results are in line with previous studies [6, 8, 9, 23, 26, 32, 35–37]. The association between increased electrolyte levels and mortality may largely reflect very high mortality rates in hypernatremic patients. Indeed, a previous study reported a mortality rate of 61% in these patients, and 50% mortality even after correction of hypernatremia[35]. Hyperkalemia has the potential for causing fatal arrhythmias especially in patients with kidney disease, cardiovascular disease, and diabetes mellitus [38]. These conditions are common and require therapeutic interventions which can induce or worsen hyperkalemia [38]. Hypercalcemia is common in patients with cancer, occurring in 20–30% of cases [39], comprising 44% of all cases of hypercalcemia in the ED [13], and is indicative of very poor prognosis; approximately 50% of hypercalcemic cancer patients die within 30 days of diagnosis [40]. The most common cause of hyperphosphatemia and hypermagnesemia is chronic kidney disease, which is particularly prevalent in older patients with other co-morbidities including cardiovascular disease [41]. Moreover, it has been shown that hyperphosphatemia is associated with increased all-cause mortality in the general population without apparent kidney disease [42]. Hyperphosphatemia can lead to adverse cardiovascular outcomes and mortality, particularly in patients with chronic kidney disease, by promoting endothelial dysfunction, vascular stiffness, and vascular calcification [42]. Hypermagnesemia, on the other hand, may cause impairment of both cardiac systolic contraction and diastolic relaxation in addition to serious arrhythmia [43].

### Limitations

Our study has limitations due to its retrospective design. We linked laboratory data to administrative data (age, sex, diagnoses, hospital LOS, readmission and mortality). The diagnoses were based on the ICD-10 coding which the responsible physician found relevant, and we did not have information concerning causes of death. We have adjusted for chronic diseases that are likely to have influenced outcomes, but there might be multiple other factors affecting LOS, readmissions and mortality which were not accounted for. Since the electronic patient journal systems in Norway are not searchable, we were unable to link the laboratory data to other potentially confounding variables, including past medical history, reason for visiting and vital status in the ED, and known history of chronic electrolyte imbalances and treatment. Although the routine at Diakonhjemmet Hospital is to take blood samples shortly after the patient has arrived in the ED, we cannot exclude that blood from some patients was collected after initiation of treatment. Furthermore, since the study was only performed in one hospital, the results could vary between centres, regions, or countries, which limits the generalizability. The results of our study regarding magnesium, free calcium, and phosphate levels appear to reflect only a subset of patients from whom these measurements were performed for clinical reasons. Thus, the true prevalence of these EIs could not be shown in this study.

## Conclusion

Our results demonstrate that EIs are common in patients admitted to an ED at a local urban hospital, and that patients with EI have an increased risk of prolonged LOS, readmission, and mortality. Thus, EIs increase consumption of health care resources. EIs may be reflect serious underlying conditions or the EI in itself may contribute to the increased risk of prolonged LOS, readmission, and mortality. It is therefore crucial that health personnel are effectively trained in the diagnosis and management of EIs. We further suggest that future studies should investigate whether an increased focus on EI detection, follow-up, and treatment can decrease the risk of prolonged LOS, readmission, and mortality.

## Supporting information

S1 Fig Flowchart and overview of analyses; Electrolyte imbalance. * LOS, length of stay.
(PNG)

S2 Fig Distribution of age categories for all visits to the emergency department 2010–2015. (PNG)

S1 Table Electrolyte imbalance categorized by sex for all ED visits 2010–2015. Abbreviations: ED, Emergency Department.
(DOCX)

S2 Table Readmissions within 30 days after discharge by degree of severity of the electrolyte imbalance for all admissions 2010–2015. Abbreviations: NA, not applicable.
(DOCX)

S3 Table Multivariate analysis for readmission, in-hospital, 30-day and 1-year mortality by degree of severity of the electrolyte imbalance for all ED visits 2010–2015. Abbreviations: ED, Emergency Department. NA, not applicable. * Adjusted for age, sex and comorbid conditions (hypertension, heart failure, atrial fibrillation/atrial flutter, chronic pulmonary disease, cancer, kidney failure, dehydration, diabetes mellitus, pneumonia, sepsis and hip fracture).
(DOCX)
