# Supplementary figures and images for: Electrolyte imbalances in an unselected population in an emergency department: A retrospective cohort study
Source: PLoS One. 2019 Apr 25;14(4):e0215673. doi: 10.1371/journal.pone.0215673 (PMC6483356; doi:10.1371/journal.pone.0215673)

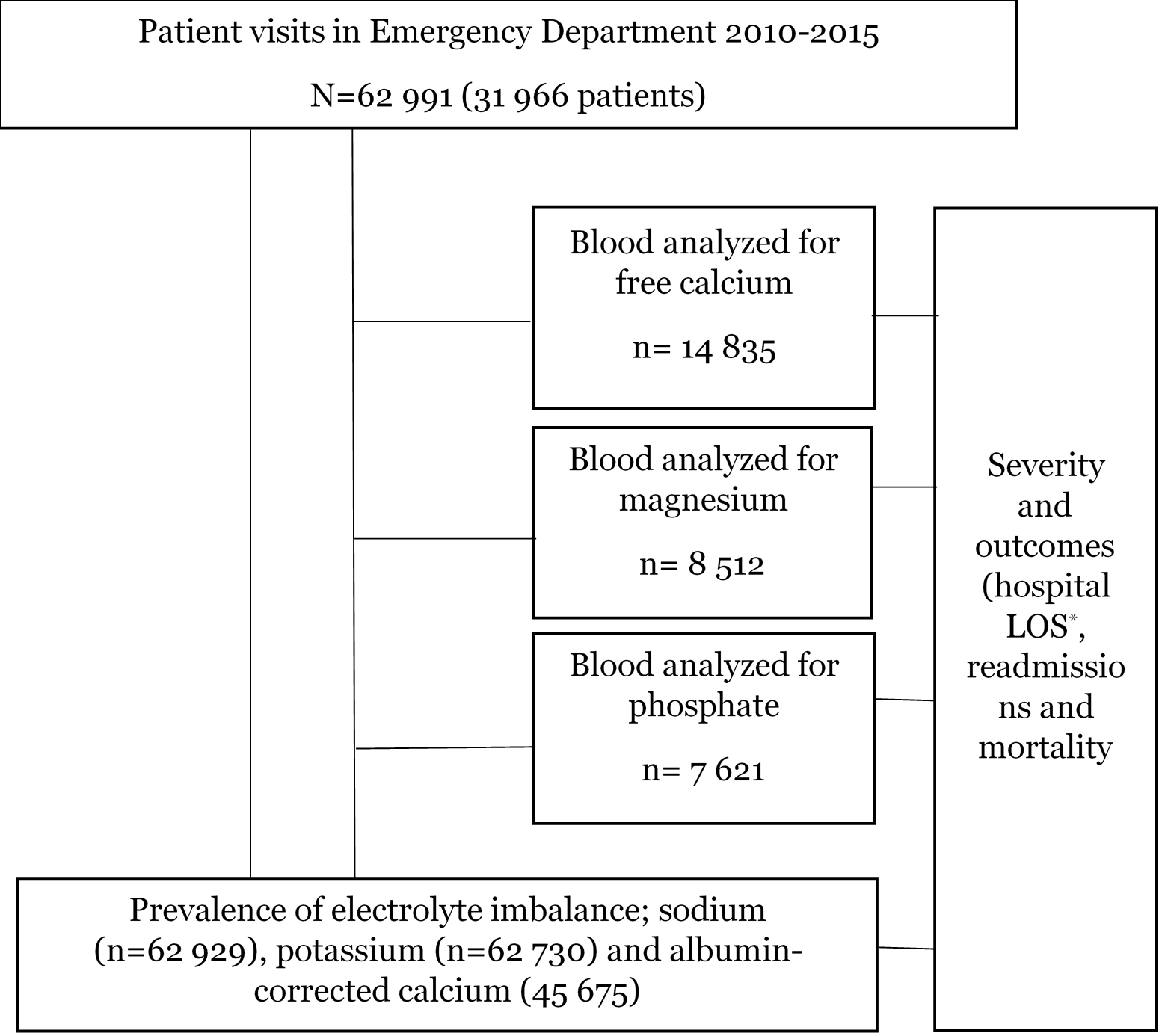

Supplement: S1 Fig — * LOS, length of stay. (PNG) [file pone.0215673.s001.png]

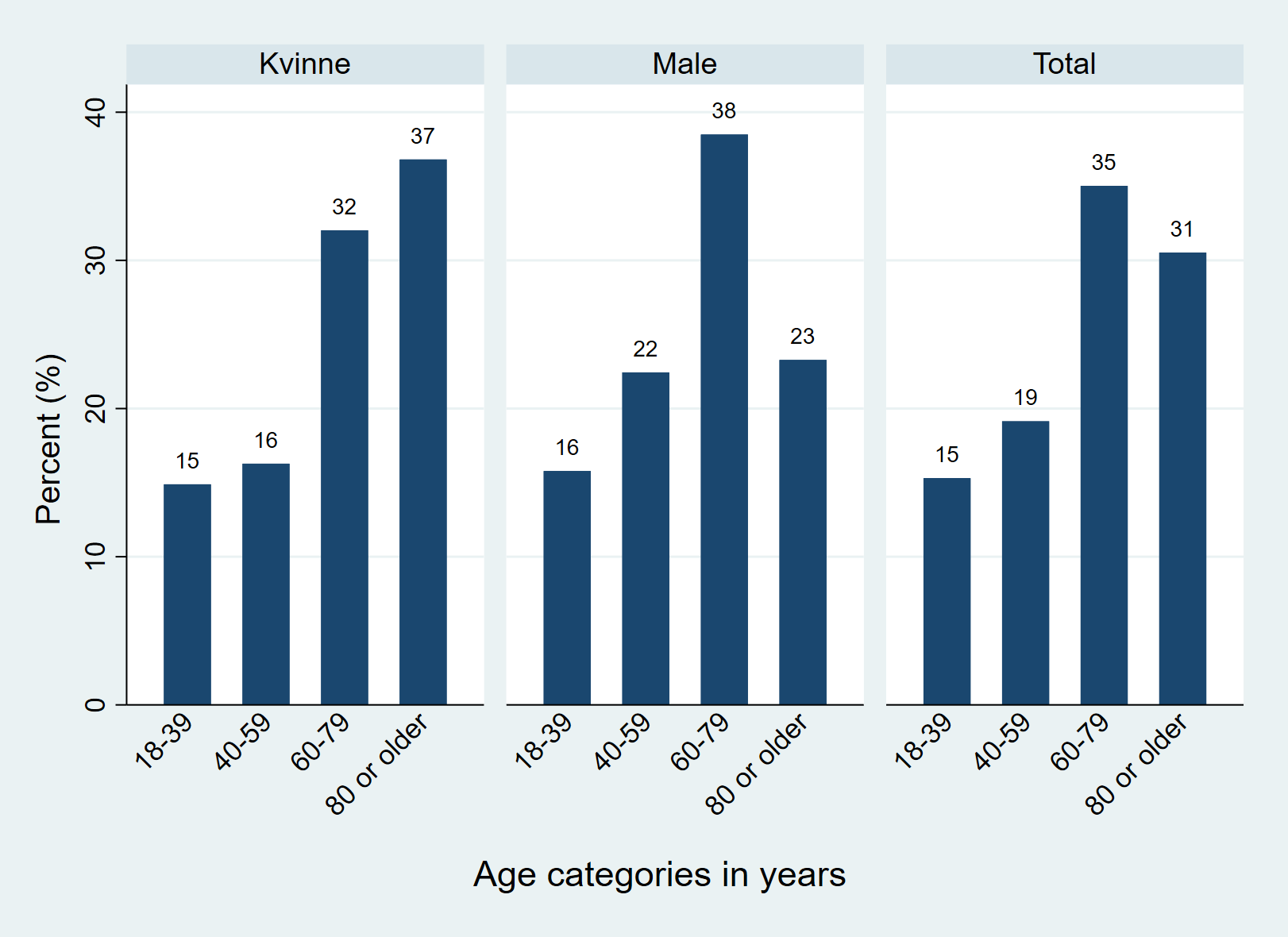

Supplement: S2 Fig — (PNG) [file pone.0215673.s002.png]
