# Supplementary material for: Electrolyte imbalances in an unselected population in an emergency department: A retrospective cohort study
Source: PLoS One. 2019 Apr 25;14(4):e0215673. doi: 10.1371/journal.pone.0215673 (PMC6483356; doi:10.1371/journal.pone.0215673)
Supplement: S1 Table — Abbreviations: ED, Emergency Department. (DOCX) [file pone.0215673.s003.docx]

| S1 Table. Electrolyte imbalance categorized by sex for all ED visits 2010-2015 | | | | |
| --- | --- | --- | --- | --- |
|  | **Female**  **n (%)** | **Male**  **n (%)** | **p-value** | **Total**  **N (%)** |
| Sodium (n=62 929) <0.001 | | | | |
| Normonatremia | 24 657 (52.7) | 22 166 (47.3) |  | 46 823 (100) |
| Hyponatremia | 8474 (56.4) | 6556 (43.6) |  | 15 030 (100) |
| Hypernatremia | 544 (50.6) | 532 (49.4) |  | 1076 (100) |
| Total | 33 675 (53.5) | 29 254 (46.5) |  | 62 929 (100) |
|  |  |  |  |  |
| Glucose-corrected sodium (n=47 467) <0.001 | | | | |
| Normonatremia | 17 593 (51.9) | 16 288 (48.1) |  | 33 881 (100) |
| Hyponatremia | 6741 (57.7) | 4951 (42.4) |  | 11 692 (100) |
| Hypernatremia | 938 (49.5) | 956 (50.5) |  | 1894 (100) |
| Total | 25 272 (53.2) | 22 195 (46.8) |  | 47 467 (100) |
| Potassium (n=62 730) <0.001 | | | | |
| Normokalemia | 29 107 (52.6) | 26 186 (47.4) |  | 55 293 (100) |
| Hypokalemia | 3585 (66.7) | 1791 (33.3) |  | 5376 (100) |
| Hyperkalemia | 880 (42.7) | 1181 (57.3) |  | 2061 (100) |
| Total | 33 572 (53.5) | 29 158 (46.5) |  | 62 730 (100) |
| Calcium (albumin-corrected) (n=45 675) <0.001 | | | | |
| Normocalcemia | 20 778 (52.0) | 19 203 (48.0) |  | 39 981 (100) |
| Hypocalcemia | 275 (38.6) | 438 (61.4) |  | 713 (100) |
| Hypercalcemia | 3237 (65.0) | 1744 (35.0) |  | 4981 (100) |
| Total | 24 290 (53.2) | 21 385 (46.8) |  | 45 675 (100) |
| Calcium (free) (n=14 835) <0.001 | | | | |
| Normocalcemia | 5618 (52.4) | 5103 (47.6) |  | 10 721 (100) |
| Hypocalcemia | 1769 (49.7) | 1790 (50.3) |  | 3559 (100) |
| Hypercalcemia | 332 (59.8) | 223 (40.2) |  | 555 (100) |
| Total | 7719 (52.0) | 7116 (48.0) |  | 14 835 (100) |
| Magnesium (n=8512) 0.002 | | | | |
| Normomagnesemia | 4084 (61.9) | 2511 (38.1) |  | 6595 (100) |
| Hypomagnesemia | 758 (61.8) | 468 (38.2) |  | 1226 (100) |
| Hypermagnesemia | 380 (55.0) | 311 (45.0) |  | 691 (100) |
| Total | 5222 (61.3) | 3290 (38.7) |  | 8512 (100) |
| Phosphate (n=7621) <0.001 | | | | |
| Normophosphatemia | 3911 (62.3) | 2362 (37.7) |  | 6273 (100) |
| Hypophosphatemia | 488 (68.2) | 227 (31.8) |  | 715 (100) |
| Hyperphosphatemia | 301 (47.6) | 332 (52.4) |  | 633 (100) |
| Total | 4700 (61.7) | 2921 (38.3) |  | 7621 (100) |

Abbreviations: ED, Emergency Department.
