# Supplementary material for: Electrolyte imbalances in an unselected population in an emergency department: A retrospective cohort study
Source: PLoS One. 2019 Apr 25;14(4):e0215673. doi: 10.1371/journal.pone.0215673 (PMC6483356; doi:10.1371/journal.pone.0215673)
Supplement: S2 Table — Abbreviations: NA, not applicable. (DOCX) [file pone.0215673.s004.docx]

| S2 Table. Readmissions within 30 days after discharge by degree of severity of the electrolyte imbalance for all admissions 2010-2015 | | | | | |
| --- | --- | --- | --- | --- | --- |
|  | **Normo-**  **n (%)** | **Mild**  **n (%)** | **Moderate**  **n (%)** | **Severe**  **n (%)** | **Total**  **n (%)** |
| Sodium | | | | | |
| Normonatremia (n=46 823) | 9169 (19.6) |  |  |  | 9169 (19.6) |
| Hyponatremia (n=15 030) |  | 3087 (20.5) | 362 (2.4) | 108 (0.7) | 3557 (23.6) |
| Hypernatremia (n=1076) |  | 175 (16.3) | 10 (0.9) | 2 (0.2) | 187 (17.4) |
| Glucose corrected sodium |  |  |  |  |  |
| Normonatremia (n=33 881)) | 6319 (18.7) |  |  |  | 6319 (18.7) |
| Hyponatremia (n= 11 692) |  | 2334 (20.0) | 276 (2.4) | 83 (0.7) | 2693 (23.1) |
| Hypernatremia (n=1894) |  | 362 (19.1) | 13 (0.7) | 6 (0.3) | 381 (20.1) |
| Potassium | | | | | |
| Normokalemia (n=55 293) | 11 352 (20.5) |  |  |  | 11 352 (20.5) |
| Hypokalemia (n=5376) |  | 919 (17.1) | 93 (1.7) | 11 (0.2) | 1023 (19.0) |
| Hyperkalemia (n=2061) |  | 452 (21.9) | 37 (1.8) | 11 (0.5) | 500 (24.2) |
| Calcium (albumin-corrected) | | | | | |
| Normocalcemia (n=39 981) | 7781 (19.5) |  |  |  | 7781 (19.5) |
| Hypocalcemia (n=713) |  | 142 (19.9) | 17 (2.4) | 5 (0.7) | 164 (23.0) |
| Hypercalcemia (n=4981) |  | 992 (19.9) | 89 (1.8) | 30 (0.6) | 1111 (22.3) |
| Calcium (free) | | | | | |
| Normocalcemia (n=10 721) | 2003 (18.7) |  |  |  | 2003 (18.7) |
| Hypocalcemia (n=3559) |  | 717 (20.2) | 20 (0.6) | 2 (0.1) | 739 (20.9) |
| Hypercalcemia (n=555) |  | 101 (18.2) | 13 (2.3) | 2 (0.4) | 116 (20.9) |
| Magnesium | | | | | |
| Normomagnesemia (n=6595) | 1193 (18.1) |  |  |  | 1193 (18.1) |
| Hypomagnesemia (n=1226) |  | 121 (9.9) | 122 (10.0) | 21 (1.7) | 264 (21.6) |
| Hypermagnesemia (n=691) |  | 133 (19.3) | NA | NA | 133 (19.3) |
| Phosphate | | | | | |
| Normophosphatemia (n=6273) | 1151 (18.4) |  |  |  | 1151 (18.4) |
| Hypophosphatemia (n=715) |  | 89 (12.5) | 30 (4.2) | 3 (0.4) | 122 (17.1) |
| Hyperphosphatemia (n=633) |  | 72 (11.4) | 15 (2.4) | 38 (6.0) | 125 (19.8) |

Abbreviations: NA, not applicable.
