# Supplementary material for: Electrolyte imbalances in an unselected population in an emergency department: A retrospective cohort study
Source: PLoS One. 2019 Apr 25;14(4):e0215673. doi: 10.1371/journal.pone.0215673 (PMC6483356; doi:10.1371/journal.pone.0215673)
Supplement: S3 Table — Abbreviations: ED, Emergency Department. NA, not applicable. * Adjusted for age, sex and comorbid conditions (hypertension, heart failure, atrial fibrillation/atrial flutter, chronic pulmonary disease, cancer, kidney failure, dehydration, diabetes mellitus, pneumonia, sepsis and hip fracture). (DOCX) [file pone.0215673.s005.docx]

| S3 Table. Multivariate analysis for readmission, in-hospital, 30-day and 1-year mortality by degree of severity of the electrolyte imbalance for all ED visits 2010-2015 | | | | | | | | |
| --- | --- | --- | --- | --- | --- | --- | --- | --- |
|  | **Multivariable analysis readmission^*^** | | **Multivariable analysis in-hospital mortality^*^** | | **Multivariable analysis 30-days mortality^*^** | | **Multivariable analysis 1-year mortality^*^** | |
|  | **Odds ratio**  **(95 % CI)** | **p-value** | **Odds ratio**  **(95 % CI)** | **p-value** | **Odds ratio**  **(95 % CI)** | **p-value** | **Hazard ratio**  **(95 % CI)** | **p-value** |
| Normonatremi (reference) | | | | | | | | |
| Mild hyponatremia | 1.18 (1.13-1.24) | <0.0001 | 1.45 (1.27-1.64) | <0.0001 | 1.23 (1.19-1.27) | <0.0001 | 1.43 (1.35-1.50) | <0.0001 |
| Moderate hyponatremia | 1.37 (1.21-1.55) | <0.0001 | 2.39 (1.83-3.13) | <0.0001 | 1.41 (1.29-1.54) | <0.0001 | 1.71 (1.50-1.95) | <0.0001 |
| Severe hyponatremia | 1.10 (0.88-1.36) | 0.409 | 2.98 (1.98-4.49) | <0.0001 | 1.30 (1.13-1.50) | <0.0001 | 1.72 (1.38-2.13) | <0.0001 |
| Mild hypernatremia | 0.82 (0.70-0.97) | 0.023 | 3.67 (2.85-4.71) | <0.0001 | 1.53 (1.38-1.71) | <0.0001 | 1.62 (1.38-1.89) | <0.0001 |
| Moderate hypernatremia | 0.76 (0.39-1.50) | 0.437 | 10.78 (5.97-19.45) | <0.0001 | 2.27 (1.36-3.78) | 0.002 | 3.03 (1.62-5.68) | 0.001 |
| Severe hypernatremia | 0.60 (0.14-2.61) | 0.493 | 14.92 (4.97-44.80) | <0.0001 | 3.59 (1.48-8.69) | 0.005 | 4.89 (1.56-15.35) | 0.007 |
| Glucose-corrected normonatremi (reference) | | | | | | | | |
| Mild hyponatremia | 1.24 (1.17-1.31) | <0.0001 | 1.39 (1.21-1.60) | <0.0001 | 1.22 (1.17-1.26) | <0.0001 | 1.41 (1.32-1.49) | <0.0001 |
| Moderate hyponatremia | 1.41 (1.22-1.62) | <0.0001 | 2.43 (1.83-3.23) | <0.0001 | 1.45 (1.32-1.59) | <0.0001 | 1.74 (1.51-2.01) | <0.0001 |
| Severe hyponatremia | 1.04 (0.81-1.32) | 0.759 | 2.91 (1.87-4.51) | <0.0001 | 1.27 (1.09-1.48) | 0.003 | 1.71 (1.35-2.16) | <0.0001 |
| Mild hypernatremia | 1.01 (0.89-1.14) | 0.869 | 3.07 (2.50-3.78) | <0.0001 | 1.36 (1.25-1.47) | <0.0001 | 1.51 (1.35-1.70) | <0.0001 |
| Moderate hypernatremia | 0.89 (0.49-1.63) | 0.710 | 12.85 (7.44-22.21) | <0.0001 | 1.99 (1.25-3.17) | 0.004 | 1.54 (0.76-3.09) | 0.228 |
| Severe hypernatremia | 1.22 (0.46-2.75) | 0.800 | 10.83 (4.54-25.80) | <0.0001 | 4.24 (2.39-7.52) | <0.0001 | 5.14 (2.54-10.37) | <0.0001 |
| Normokalemia (reference) | | | | | | | | |
| Mild hypokalemia | 0.90(0.84-0.98) | 0.010 | 1.62 (1.35-1.96) | <0.0001 | 1.17 (1.11-1.23) | <0.0001 | 1.21 (1.11-1.32) | <0.0001 |
| Moderate hypokalemia | 1.14 (0.90-1.45) | 0.265 | 2.94 (1.88-4.60) | <0.0001 | 1.56 (1.35-1.81) | <0.0001 | 1.45 (1.15-1.83) | 0.002 |
| Severe hypokalemia | 1.29 (0.65-2.55) | 0.468 | 3.99 (1.24-12.80) | 0.020 | 3.36 (2.06-5.49) | <0.0001 | 5.28 (1.84-9.84) | <0.0001 |
| Mild hyperkalemia | 1.11 (0.99-1.24) | 0.063 | 2.93 (2.43-3.53) | <0.0001 | 1.45 (1.35-1.56) | <0.0001 | 1.54 (1.39-1.71) | <0.0001 |
| Moderate hyperkalemia | 0.84 (0.58-1.22) | 0.364 | 7.30 (4.92-10.84) | <0.0001 | 1.39 (1.10-1.76) | 0.006 | 1.52 (1.08-2.15) | 0.017 |
| Severe hyperkalemia | 1.16 (0.59-2.30) | 0.671 | 4.28 (1.66-11.04) | 0.003 | 1.75 (1.10-2.79) | 0.018 | 1.45 (0.72-2.92) | 0.295 |
| Normocalcemia (albumin-corrected calcium, reference) | | | | | | | | |
| Mild hypocalcemia | 1.13 (0.94-1.38) | 0.193 | 1.27 (0.80-2.00) | 0.312 | 1.37 (1.21-1.57) | <0.0001 | 1.41 (1.16-1.71) | 0.001 |
| Moderate hypocalcemia | 2.66 (1.39-5.10) | 0.003 | 4.32 (1.55-12.05) | 0.005 | 2.69 (1.75-4.12) | <0.0001 | 3.51 (2.15-5.75) | <0.0001 |
| Severe hypocalcemia | 1.83 (0.61-5.45) | 0.278 | NA | NA | 1.31 (0.59-2.92) | 0.510 | 1.62 (0.67-3.90) | 0.282 |
| Mild hypercalcemia | 1.05 (0.97-1.13) | 0.249 | 2.27 (1.96-2.62) | <0.0001 | 1.42 (1.36-1.49) | <0.0001 | 1.65 (1.53-1.77) | <0.0001 |
| Moderate hypercalcemia | 1.26 (0.98-1.62) | 0.067 | 3.08 (2.13-4.46) | <0.0001 | 1.61 (1.37-1.89) | <0.0001 | 2.27 (1.84-2.81) | <0.0001 |
| Severe hypercalcemia | 1.12 (0.73-1.72) | 0.611 | 2.98 (1.65-5.38) | <0.0001 | 2.22 (1.68-2.94) | <0.0001 | 2.39 (1.66-3.43) | <0.0001 |
| Normocalcemia (free-calcium) | | | | | | | | |
| Mild hypocalcemia | 1.10 (1.00-1.22) | 0.046 | 1.08 (0.91-1.30) | 0.374 | 1.09 (1.02-1.16) | 0.011 | 1.13 (1.03-1.25) | 0.013 |
| Moderate hypocalcemia | 1.18 (0.71-1.96) | 0.512 | 2.00 (0.93-4.30) | 0.077 | 1.53 (1.10-2.15) | 0.012 | 1.45 (0.87-2.42) | 0.151 |
| Severe hypocalcemia | 1.72 (0.34-8.66) | 0.510 | NA | NA | 0.79 (0.11-5.61) | 0.812 | 1.65 (0.23-11.75) | 0.617 |
| Mild hypercalcemia | 0.95 (0.76-1.20) | 0.673 | 2.22 (1.64-3.00) | <0.0001 | 1.44 (1.25-1.66) | <0.0001 | 1.66 (1.36-2.02) | <0.0001 |
| Moderate hypercalcemia | 1.65 (0.84-3.28) | 0.148 | 1.43 (0.52-3.94) | 0.485 | 1.18 (0.70-2.01) | 0.530 | 1.31 (0.68-2.54) | 0.422 |
| Severe hypercalcemia | 0.65 (0.14-3.04) | 0.583 | 1.95 (0.36-10.58) | 0.441 | 1.39 (0.57-3.35) | 0.464 | 0.54 (0.07-3.85) | 0.539 |
| Normomagnesemia (reference) | | | | | | | | |
| Mild hypomagnesemia | 1.16 (0.94-1.44) | 0.168 | 1.16 (0.77-1.75) | 0.478 | 1.07 (0.94-1.21) | 0.264 | 1.10 (0.90-1.34) | 0.349 |
| Moderate hypomagnesemia | 1.25 (1.01-1.54) | 0.043 | 0.99 (0.62-1.59) | 0.988 | 1.15 (1.02-1.30) | 0.022 | 1.40 (1.16-1.70) | <0.0001 |
| Severe hypomagnesemia | 1.95 (1.16-3.30) | 0.012 | NA | NA | 1.42 (0.99-2.01) | 0.052 | 1.99 (1.26-3.15) | 0.003 |
| Mild hypermagnesemia | 1.11 (0.90-1.36) | 0.338 | 2.47 (1.85-3.32) | <0.0001 | 1.14 (1.00-1.28) | 0.037 | 1.27 (1.06-1.52 | 0.009 |
| Moderate hypermagnesemia | NA | NA | NA | NA | NA | NA | NA | NA |
| Severe hypermagnesemia | NA | NA | NA | NA | NA | NA | NA | NA |
| Normophosphatemia (reference) | | | | | | | | |
| Mild hypophosphatemia | 0.91 (0.72-1.16) | 0.446 | 0.94 (0.59-1.50) | 0.798 | 0.94 (0.83-1.06) | 0.281 | 0.76 (0.61-0.95) | 0.018 |
| Moderate hypophosphatemia | 0.99 (0.66-1.48) | 0.963 | 0.51 (0.19-1.37) | 0.183 | 0.93 (0.74-1.17) | 0.525 | 0.75 (0.49-1.15) | 0.192 |
| Severe hypophosphatemia | 2.49 (0.58-10.7) | 0.221 | NA | NA | 1.45 (0.54-3.89) | 0.455 | 1.61 (0.22-11.47) | 0.634 |
| Mild hyperphosphatemia | 1.07 (0.82-1.41) | 0.604 | 3.27 (2.27-4.70) | <0.0001 | 1.40 (1.19-1.65) | <0.0001 | 1.55 (1.23-1.95) | <0.0001 |
| Moderate hyperphosphatemia | 0.68 (0.39-1.18) | 0.173 | 4.46 (2.58-7.71) | <0.0001 | 0.95 (0.68-1.32) | 0.761 | 0.73 (0.42-1.27) | 0.265 |
| Severe hyperphosphatemia | 1.43 (0.97-2.12) | 0.073 | 4.84 (2.99-784) | <0.0001 | 1.30 (0.99-1.71) | 0.052 | 1.36 (0.94-1.98) | 0.100 |

Abbreviations: ED, Emergency Department. NA, not applicable.

^*^ Adjusted for age, sex and comorbid conditions (hypertension, heart failure, atrial fibrillation/atrial flutter, chronic pulmonary disease, cancer, kidney failure, dehydration, diabetes mellitus, pneumonia, sepsis and hip fracture).
